# Seamless multistage laser-plasma acceleration toward future high-energy colliders

**DOI:** 10.1038/s41377-018-0037-6

**Published:** 2018-06-20

**Authors:** Kazuhisa Nakajima

**Affiliations:** High Energy Accelerator Research Organization, Tsukuba, 305-0801 Japan

**Multistage laser wakefield accelerators that are coupled with variable-curvature plasma channels make it possible to efficiently accelerate electrons to high energies that exceed dephasing and pump depletion limits. Seamless coupling between laser and particle beams may envisage future energy-frontier colliders of revolutionarily small size and cost.**

In the long-standing quest for the fundamental building blocks of nature, in the so-called Standard Model of particle physics, energy-frontier colliders have played a central role in the forefront of research on matter and interactions. For future high-energy particle colliders to explore physics beyond the Standard Model, a proton–proton circular collider at an energy of 100 TeV and a circumference of 100 km or an electron–positron linear collider with an energy that is on the order of 1 TeV and a length of 30 km are being considered around the world, exploiting conventional technologies such as superconducting magnets and RF systems^1^. In contrast to proton colliders, which create clouds of debris, electron–positron colliders enable cleaner and more precise experiments on fundamental particle collisions. Currently, a diverse collection of electron–positron linear colliders is proposed for the application of advanced accelerator concepts^2^, such as two-beam accelerators (TBAs), dielectric-wakefield accelerators (DWAs), beam-driven plasma wakefield accelerators (PWFAs), and laser-driven plasma wakefield accelerators (LWFAs), which promise a much higher accelerating gradient than that of a conventional RF accelerator. The feasibility of particle colliders based on advanced technologies depends on not only downsizing the machine but also reducing the project cost, including the construction and electrical power costs, to satisfy the collider performance requirements. In a recent publication, Luo and coworkers proposed a compact and efficient scheme of multistage laser wakefield accelerators coupled with specially designed curved plasma channels for feeding fresh laser pulses to straight acceleration stages. They presented particle-in-cell (PIC) simulation results that demonstrate successive acceleration of high-quality electron beams through a coupling segment^3^.

In LWFAs^4^, an intense, short laser pulse propagating in plasma with an electron density of *n*_*e*_ cm^−3^ can generate a plasma wakefield on the order of ~*n*_*e*_^1/2^ V/cm due to the density oscillation that is excited by the ponderomotive force of the laser pulse, which is the average nonlinear force that is exerted on plasma electrons by the laser field. While LWFAs provide enormous accelerating gradients of as high as 1 GV/cm^−1^ at a plasma density of 10^18^ cm^−3^, the dephasing of relativistic electrons with respect to a correct acceleration phase of the plasma wakefield with a phase velocity that is smaller than the speed of light in vacuum and energy depletion of the laser pulse limit the electron energy gain in a single stage. A straightforward solution for overcoming the dephasing and pump depletion effects is to build a multistage accelerator that is composed of consecutive LWFA stages such that the final energy gain reaches the required beam energy without loss of the beam charge and qualities through a coupling segment where a fresh laser pulse is fed to continuously accelerate the electron beam from the previous stage.

Since the electron beam size with a finite beam emittance causes a rapid growth in the vacuum drift space outside plasma^5^, the coupling segment must be used for spatial matching of the electron beam with the transverse wakefield and temporal phase matching with the accelerating wakefield in a subsequent stage. The total length of a multistage LWFA can be minimized by choosing the coupling distance to be equal to half of the dephasing length, which leads to a staging length of 1.5 times the dephasing length, using either the conventional coupling scheme or a plasma-based scheme, as proposed in ref. ^6^. Assuming that the plasma-based technologies reach a high level of maturity, the plasma-based coupling scheme that consists of a plasma lens for the electron beam injection and a plasma mirror or a curved plasma channel for the laser pulse injection makes it possible to shorten the coupling distance to the order of 10 cm at an operating plasma density of 10^17^ cm^−3^, compared to the conventional scheme, which consists of a magnet-based beam focusing system and a laser focusing mirror system that require a coupling distance that is on the order of 10 m at a final beam energy of 1 TeV ^6^.

A proof-of-principle experiment on two LWFA stages powered by two synchronized laser pulses through the plasma lens and mirror coupling has been reported^7^. It is shown that a 120-MeV electron beam from a gas jet (the first stage) driven by a 28-TW, 45-fs laser pulse was focused by a first discharge capillary as an active plasma lens to a second capillary plasma channel (the second stage), where the wakefield that was excited by a 12-TW, 45-fs separate laser pulse reflected by a tape-based plasma mirror with a laser-energy throughput of 80% further increased the energy gain to 100 MeV. In this experiment, the trapping fraction of the electron charge coupled to the second stage was as low as 3.5%. However, a 100% trapping efficiency with multistage coupling is required in high-energy accelerators.

In this context, such a poor coupling efficiency could be attributed to the plasma mirror inserted at a vacuum drift space, where an electron bunch cannot be transported within a sufficiently strong focusing channel to preserve the phase-space matching of betatron motion with subsequent wakefields. Luo’s proposed multistage coupling scheme, which uses a variable curvature plasma channel, enables off-axial injection of a fresh laser pulse into the LWFA stage without a vacuum gap in the coupling segment. Thus, an electron bunch will be continuously accelerated through the plasma focusing channel over the consecutive stages only if the temporal phase-matching between laser and electron beams is optimized. This is akin to a conventional accelerating structure that is composed of an RF cavity and a coupler.

**Fig. 1:**
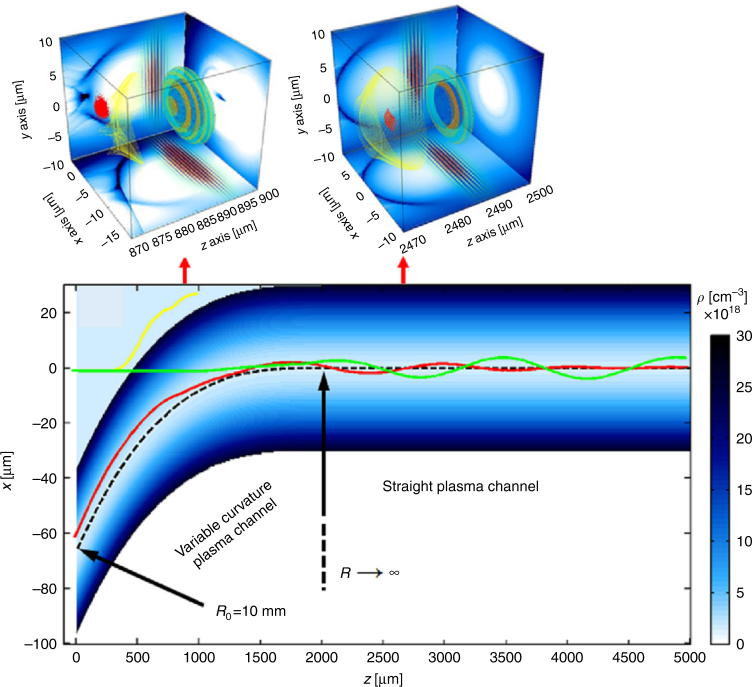
Geometry of the coupling segment, which is composed of a variable-curvature plasma channel with a gradually varying channel radius along the channel axis (dashed line) from the entrance radius *R*_*0*_ = 10 mm in the first stage to that of a straight channel *R* → ∞ in the second stage, and the centroid trajectories for a first-stage laser (yellow), a second-stage laser (red), and an electron beam (green). When the second laser is injected at the curved channel entrance with an incidence angle of 5.7° and an off-axis deviation of 6.3 μm, its centroid trajectory and an electron bunch (red points) are seamlessly coupled to the straight plasma channel, as shown in the 3D PIC simulation results before and after the electron bunch is trapped in the second straight channel

In a laser guiding experiment that uses a curved discharge capillary with a radius of curvature of 10 cm, 85% transmission of a laser pulse at the intensity of 10^16^ W/cm^2^ has been observed^8^. In the propagation of a laser pulse through a curved plasma channel, the radial equilibrium position of the laser pulse is shifted away from the channel axis due to the balance between the refractive index gradient bending the light rays inward and the centrifugal force pulling them outward. As a result, the minimum effective plasma density, which is proportional to a guiding potential, is located outside the channel axis^9^. Thus, the direct guiding of a laser pulse from the curved channel with constant curvature to the straight channel causes large centroid oscillations in the straight channel, even though the laser pulse is injected at the equilibrium position, leading to loss of the laser energy and electron beam transported from the previous stage as a result of the off-axis interaction with the wakefield. To diminish the mismatch at the transition from a curved channel to a straight one, Luo and coworkers devised a variable-curvature plasma channel such that the equilibrium position guides the laser centroid gradually along the channel axis from the initial equilibrium position to the channel center, where the straight channel axis merges with that of the curved channel, as shown in Fig. 1. A seamless acceleration in two-stage LWFA coupled with a variable-curvature plasma channel was successfully demonstrated for the guided laser intensity of 8.55 × 10^18^ W/cm^2^ (normalized vector potential of 2) and a nonlinear wakefield in the bubble regime in the 3D PIC simulations (Fig. 1), and it was shown that the injection trapping efficiency increases with the initial beam energy and approaches 100% at energies that are higher than 2 GeV.

Toward an application for electron–positron colliders, it will be necessary in the next step for the multistage acceleration of a positron bunch through the proposed coupling scheme to be examined in detail for the linear and nonlinear LWFA regimes, along with the experimental demonstration of a seamless coupling using variable-curvature plasma channels.
